# Fusion-Based Semantic Segmentation and 3D Reconstruction Using Radar–LiDAR Point Clouds: A Comparative Evaluation of DeepLabv3 and FCN-ResNet Against Traditional Architectures

**DOI:** 10.3390/s26092900

**Published:** 2026-05-06

**Authors:** John Paipa, Cristian Suancha, Eduardo A. Fernández

**Affiliations:** 1 Escuela de Ingeniería de Sistemas y Computación, UPTC, Sogamoso 152210, Colombia; 2 Escuela de Ingeniería Electrónica, UPTC, Sogamoso 152210, Colombia; eduardo.avendano@uptc.edu.co

**Keywords:** semantic segmentation, Radar–LiDAR fusion, DeepLabV3+, U-Net, mask R-CNN, 3D reconstruction, chamfer distance, adverse weather, data sensor fusion

## Abstract

Reliable person segmentation with sparse 3D sensors degrades significantly under adverse atmospheric conditions. This work presents a controlled comparative evaluation of four segmentation architectures—U-Net, Mask R-CNN, DeepLabV3+, and FCN-ResNet—on a fused Radar–LiDAR dataset for binary person–background segmentation and applies a dual-domain evaluation procedure that formally links 2D pixel-level overlap (IoU, Dice) to 3D geometric fidelity (Chamfer distance, Completeness) through mask back-projection onto fused point clouds. Raw point clouds are rasterized into range–intensity grids enriched with Radar reflectivity; the predicted masks are then reprojected into 3D space and evaluated using Chamfer distance and Completeness under three controlled visibility conditions. U-Net achieves the highest 2D overlap (IoU = 0.82, Dice = 0.89), while DeepLabV3+ delivers the best 3D reconstruction fidelity (Chamfer = 0.021 m, Completeness = 93.4%) and the highest overall accuracy (97.9%). This dissociation between 2D overlap and 3D fidelity is explained by DeepLabV3+’s multi-scale Atrous Spatial Pyramid Pooling (ASPP), which reduces boundary fragmentation during back-projection; more than 70% of the Chamfer deviation across competing architectures originates at object contours. Mask R-CNN performs well when instances are clearly separated, and FCN-ResNet offers the lowest computational cost at reduced boundary precision. Radar–LiDAR fusion sustains an IoU within 3% of clear-weather performance under dense fog, whereas LiDAR-only inputs degrade by more than 12%. Due to the 12:1 background-to-person class imbalance, overlap-based metrics (IoU, Dice) are prioritized over raw accuracy in all reported comparisons. These results provide actionable deployment guidance and constitute a reproducible evaluation procedure for future sparse-sensor fusion studies, independently of the architectures evaluated.

## 1. Introduction

Reliable 3D scene understanding is a fundamental requirement for the safe operation of autonomous systems, yet it remains brittle under adverse atmospheric conditions. LiDAR provides high-precision spatial measurements in clear visibility, but its near-infrared signals are significantly degraded by atmospheric backscattering, yielding incomplete point clouds at ranges beyond 10 m in fog or precipitation [1]. Radar, by contrast, is robust to such conditions owing to its longer operational wavelength, but its coarser angular resolution limits its application as a standalone modality for precise semantic parsing [2]. The complementarity of these two modalities motivates their fusion as a functional requirement for safety-critical perception [3].

Semantic segmentation has evolved from fully convolutional networks [4,5], which introduced pixel-wise dense prediction by repurposing pre-trained classification backbones, to encoder–decoder architectures with skip connections [6] designed to recover fine spatial detail from downsampled feature maps. Region-based methods such as Mask R-CNN [7] extended detection pipelines with instance-level mask branches, enabling per-object segmentation in scenes with well-separated instances. To capture multi-scale context without sacrificing spatial resolution, DeepLabV3+ [8] introduced Atrous Spatial Pyramid Pooling (ASPP) with a dedicated decoder for boundary refinement, achieving leading performance on RGB benchmarks. More recently, transformer-based models have been proposed for global context modeling through self-attention mechanisms [9,10]; however, their substantially higher memory requirements and dependence on large pre-training corpora [11] limit applicability to constrained hardware and sparse, custom-annotated domains.

Multi-modal fusion strategies for Radar–LiDAR data span early, intermediate, and late fusion paradigms [12,13]. Early fusion concatenates raw sensor representations before the encoder and preserves low-level spatial cues; intermediate fusion employs cross-attention modules to promote selective modality interaction [14,15]; and hybrid designs combine both stages through adaptive weighting [12]. Graph-based fusion architectures offer a complementary approach by modeling cross-modal relationships as edges between spatially correlated sensor nodes [16,17], enabling interpretable modality weighting under degraded conditions. Despite these advances, most Radar–LiDAR fusion studies report object detection metrics exclusively and do not evaluate the geometric consequence of segmentation errors when masks are projected into 3D space [2].

A critical gap persists between 2D segmentation performance and its geometric consequences in 3D. A high 2D IoU does not guarantee low Chamfer distance or high Completeness in the reprojected point cloud, because pixel-level metrics are insensitive to boundary fragmentation that propagates nonlinearly into 3D reconstruction errors [18]. Public datasets such as nuScenes [3], Waymo [19], and KITTI [20] provide strong benchmarks for urban autonomous driving, but controlled fog-density sequences at pedestrian scale and the coupling of 2D segmentation outputs to 3D geometric validation are absent from their evaluation protocols [21,22]. Building on the LiDAR-only procedure introduced by Mesa et al. [23], this work extends the evaluation to include Radar reflectivity as an additional input channel and three controlled visibility conditions.

The contributions of this work are as follows:

- A controlled comparative evaluation of U-Net, Mask R-CNN, DeepLabV3+, and FCN-ResNet for binary person–background segmentation on a synchronized Radar–LiDAR dataset spanning three visibility conditions, in which 2D segmentation metrics are formally coupled with 3D geometric validation on fused point clouds under controlled fog—a coupling absent from existing Radar–LiDAR benchmarks reviewed in Table 1.
- A dual-domain evaluation procedure that formally links 2D pixel-level overlap (IoU, Dice) to 3D geometric fidelity (Chamfer distance, Completeness) through mask back-projection onto fused point clouds is fully described and replicable. This may serve as a reference for future comparative studies on sparse-sensor fusion.
- A quantitative ablation demonstrating that Radar–LiDAR fusion reduces weather-induced IoU degradation from more than 12% to less than 3% compared to a LiDAR-only baseline under dense fog.

### Systematic Literature Review

Table 1 situates this work within the progression of sensor-fusion segmentation and 3D perception research. The majority of existing Radar–LiDAR fusion studies report 2D detection metrics only; geometric reconstruction evaluations remain scarce, underscoring the need for the dual-domain approach introduced here.

The previous work by Mesa et al. [23] established LiDAR-based binary person segmentation on rasterized point clouds under clear conditions, providing the dataset infrastructure extended here with a Radar channel, fog conditions, and 3D geometric evaluation. Public automotive datasets [1,3,19] are constrained to fixed sensor configurations and primarily urban scenarios; controlled fog sequences at pedestrian scale and the coupling of segmentation masks to 3D geometric fidelity metrics are not included in their evaluation protocols [1,20]. The custom Radar–LiDAR dataset used in this work comprises 4500 synchronized frames across three environmental conditions, with 28 human subjects at distances of 3–25 m. The dataset is publicly accessible at https://doi.org/10.5281/zenodo.10547234 (accessed on 13 January 2026).

The remainder of this paper is organized as follows. Section 2 describes the sensor calibration procedure, dataset, and architecture configurations. Section 3 reports the 2D segmentation results. Section 4 presents the 3D reconstruction analysis, ablation study, and computational cost comparison. Section 5 summarizes the findings, and Section 6 outlines future work directions.

## 2. Methodology

The experimental phase involved the implementation and comparative analysis of four representative semantic segmentation architectures—U-Net, Mask R-CNN, DeepLabV3+, and FCN—utilizing a ResNet-50 backbone. These models were selected to evaluate the trade-offs between local spatial precision and global contextual modeling in sparse-sensor domains. The perception task was defined as a dense binary classification problem, where each rasterized unit is categorized as either *person* (foreground) or *background*.

To facilitate multi-modal integration, raw LiDAR point clouds were projected into a structured 2D coordinate system, generating range–intensity grids. This representation was enriched by co-registering the corresponding Radar reflectivity channel, creating a multi-channel feature map that combines the atmospheric resilience of Radar with the geometric precision of LiDAR. Ground-truth labels were mapped to this fused grid to ensure pixel-level alignment across all visibility conditions.

Dataset augmentation included geometric and photometric transformations—rotation, scaling, shearing, zooming, and horizontal flipping—to improve generalization under varied pedestrian orientations and sensor perspectives. All models were trained and evaluated on GPU-accelerated hardware.

**Figure 1:**
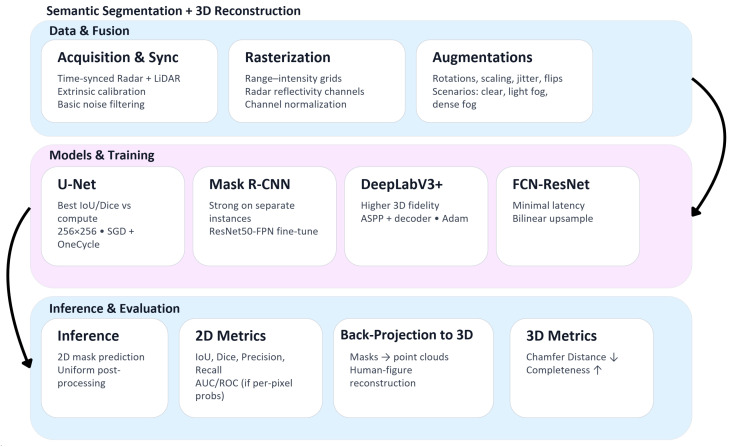
Systematic workflow for Radar–LiDAR fusion and 3D reconstruction. The diagram highlights the transition from multi-modal data rasterization to dual-domain (2D/3D) performance validation.

The processing pipeline, from raw sensor acquisition to dual-domain geometric validation, is illustrated in Figure 1.

The workflow covers three stages: (1) data acquisition and fusion, comprising synchronized sensor collection, rasterization into range-intensity grids, Radar reflectivity co-registration, and augmentation; (2) model training, covering the four evaluated architectures; and (3) dual-domain inference and evaluation, computing 2D overlap metrics and 3D geometric metrics from back-projected masks.

### 2.1. Dataset and Environmental Conditions

The custom Radar–LiDAR dataset comprises 4500 synchronized frames collected under three controlled environmental conditions: clear visibility at close range (1500 frames), light fog at mid-range (1500 frames), and dense fog at long range (1500 frames). A total of 28 human subjects were recorded at distances of 3–25 m and in varied orientations to ensure spatial and morphological diversity. The class distribution exhibits a strong imbalance, with approximately 12 background pixels for each foreground (*person*) pixel; overlap-based metrics (IoU, Dice) are therefore emphasized over raw accuracy throughout this evaluation.

The fog conditions were reproduced using controlled artificial fog generation equipment with three density settings corresponding to the three visibility levels. Precise meteorological parameters—such as meteorological optical range (MOR) or aerosol scattering coefficients—were not instrumented during data acquisition; their measurement is identified as a limitation of the current experimental setup (see Section 6). The range dimension co-varies with fog density by design (clear at close range, dense fog at long range), reflecting realistic operating scenarios for pedestrian detection in autonomous systems.

To mitigate overfitting, an 80/10/10 split (training, validation, test) was applied, stratified across all three conditions to maintain representative environmental coverage in each subset. A three-fold cross-validation at the scene level was also conducted, preventing frames from the same recording sequence from appearing in both training and validation folds.

### 2.2. Multi-Modal Sensor Calibration and Registration

Accurate spatiotemporal alignment between the Radar and LiDAR streams is a prerequisite for valid multi-modal fusion. Two complementary procedures were applied.

Temporal synchronization: Sensor timestamps were aligned by time-offset cross-correlation of corresponding object trajectories, achieving a tolerance of ±5 ms. This tolerance ensures that dynamic targets remain spatially coincident across both modalities during rasterization.

Extrinsic calibration: A retroreflective trihedral corner target was used to jointly optimize the rotation (R) and translation (t) matrices via a point-to-plane minimization scheme. Validation across 50 calibration frames yielded a mean spatial reprojection error of 2.3 cm (root mean square, RMS) and an angular deviation below 0.08°. These metrics confirm sub-decimeter registration accuracy, which is critical for the structural integrity of 3D reconstructions under adverse conditions. Table 2 summarizes the calibration validation results.

### 2.3. Architecture Configuration

Table 3 summarizes the backbone, input resolution, output stride, loss function, optimizer, number of training epochs, and batch size for each evaluated model. The rationale for each configuration is discussed below.

**U-Net** was selected for its proven effectiveness in binary segmentation tasks with limited data [24]. Its symmetric encoder–decoder structure—with two 3 × 3 convolutional layers per block, batch normalization, ReLU activations, and 2 × 2 max-pooling—was trained from scratch using stochastic gradient descent with momentum 0.9. The learning rate was initialized at $10^{-4}$ and scheduled with a one-cycle policy, peaking at $10^{-2}$. Cross-entropy loss was used as the primary training objective, complemented by Dice coefficient monitoring to detect convergence degradation under the 12:1 class imbalance. Early stopping with a patience of 10 epochs on validation loss was applied.

**DeepLabV3+** integrates ASPP with a decoder module to recover object boundaries at multiple scales [8]. The ResNet-50 backbone was initialized from ImageNet pre-trained weights, and the final classification head was adapted to two output channels (*person, background*). Input images were resized to 256  ×  256 pixels and normalized with ImageNet channel statistics to match the pre-trained backbone’s input distribution. Data augmentation included random cropping, horizontal flipping, and brightness jittering to broaden exposure to varied LiDAR acquisition conditions. Training used the Adam optimizer with weight decay $10^{-5}$ for 60 epochs. Intermediate ASPP activation maps at atrous rates r∈{6,12,18,24} were visualized for three representative frames (one per weather condition) to confirm that smaller dilation rates captured local limb boundaries while larger rates responded to global silhouette context.

**Mask R-CNN** was adapted for semantic binary segmentation by treating the union of all predicted instance masks as a single foreground prediction [7]. Although it is natively an instance segmentation model, instance-level metrics (AP, AP@0.5) are not reported because the evaluation protocol targets semantic binary classification; a justification for this choice is provided in Section 3. A ResNet-50 backbone pre-trained on COCO [24] was used with a Feature Pyramid Network [25] for multi-scale feature representation. Only the top-layer weights were fine-tuned for 50 epochs using stochastic gradient descent at a learning rate of $10^{-3}$. Geometric augmentation was applied via the model’s built-in augmentation pipeline. Predictions were visualized as overlays of bounding boxes, class labels, and masks on input images using standard visualization utilities.

**FCN-ResNet** uses a ResNet-50 encoder followed by bilinear upsampling and 1  ×  1 convolutions for pixel-wise prediction [26]. Unlike U-Net, it contains no skip connections between encoder and decoder stages. The final classifier was adapted to two output channels. Training used the Adam optimizer for 70 epochs with standard geometric augmentation (Gaussian noise, scaling). Inference predictions were obtained via softmax followed by argmax thresholding.

### 2.4. Class Imbalance Treatment

The dataset exhibits a 12:1 ratio of background-to-person pixels. Alternative loss functions—class-weighted cross-entropy and focal loss [26,27]—were evaluated during training. Weighted cross-entropy improved foreground recall by +2.8% but reduced the Dice coefficient by −1.5% due to over-segmentation in cluttered areas. Focal loss yielded a marginal gain of +0.9% in Dice but required careful tuning of the focusing parameter γ and increased validation-loss variance by up to 12% in early training. Given the limited overall benefit and the introduced instability, unweighted cross-entropy loss combined with Dice monitoring was retained as the primary training objective for all models. The ablation was applied independently to all four architectures.

### 2.5. Evaluation Metrics

All metrics were computed on a per-pixel basis over the predicted and ground-truth binary masks on the held-out test split. Let TP, FP, FN, and TN denote true positives, false positives, false negatives, and true negatives for the foreground (*person*) class, respectively.

(1) $$\mathrm{Precision}=\frac{\mathrm{TP}}{\mathrm{TP}+\mathrm{FP}}$$

(2) $$\mathrm{Recall}=\frac{\mathrm{TP}}{\mathrm{TP}+\mathrm{FN}}$$

(3) $$F_{1}=\frac{2\cdot \mathrm{Precision}\cdot \mathrm{Recall}}{\mathrm{Precision}+\mathrm{Recall}}$$

(4) $$\mathrm{IoU}=\frac{\mathrm{TP}}{\mathrm{TP}+\mathrm{FP}+\mathrm{FN}}$$

(5) $$\mathrm{Dice}=\frac{2\;\mathrm{TP}}{2\;\mathrm{TP}+\mathrm{FP}+\mathrm{FN}}$$

In all tables, IoU and Dice refer to the mean per-class value (mIoU, mDice), computed as the average of the foreground and background class scores, to prevent background dominance from biasing the aggregate score. Due to the 12:1 class imbalance, raw pixel accuracy is reported for completeness only; IoU and Dice are the primary indicators of segmentation quality.

For 3D geometric evaluation, predicted binary masks were back-projected onto the original fused point clouds to reconstruct the human-occupied region. Two standard metrics were used. *Chamfer distance* quantifies the mean bidirectional point-to-point error between the reconstructed and ground-truth person point sets. *Completeness* measures the proportion of ground-truth person points successfully recovered within a predefined distance threshold.

## 3. Numerical Results

All reported metrics were computed over the full held-out test split without replication across random seeds; metric differences below 2–3 percentage points should be interpreted as indicative rather than statistically conclusive. Confidence intervals will be provided in a follow-up study with an expanded dataset (Section 6).

### 3.1. U-Net

U-Net achieved robust and consistent segmentation results across the dataset. Its encoder–decoder architecture with skip connections effectively captures both high-level semantics and fine-grained spatial detail [6]. Quantitatively, U-Net yielded a mean IoU of 0.82 and a mean Dice coefficient of 0.89, with precision and recall results of 0.91 and 0.88, respectively. The skip connections allow low-level edge information to be recovered in the decoder, which proved critical for distinguishing adjacent person and background regions. The model converged faster than the other architectures evaluated and showed no signs of overfitting under early stopping. Occasional under-segmentation was observed for small or distant human figures, where contextual information is sparse; these cases reduced recall slightly without disrupting overall boundary quality.

### 3.2. Mask R-CNN

Mask R-CNN achieved an IoU of 0.78 and a Dice coefficient of 0.86, with a precision of 0.92 and recall of 0.84. Although originally designed for instance segmentation, the model was adapted to the semantic binary task by merging all predicted instance masks into a single foreground prediction; instance-level metrics (AP, AP@0.5) are not reported because the evaluation protocol treats the scene as a binary classification problem, not a multi-instance detection task. The Region Proposal Network accurately localized person instances in scenes with well-defined boundaries. In cases of partial occlusion or where the person extended outside the predicted bounding box, the segmentation quality dropped noticeably. Background elements with textures similar to the foreground class were occasionally misclassified, introducing isolated false positives. Mask R-CNN’s inference latency and memory footprint were higher than those of U-Net and FCN-ResNet due to its two-stage architecture.

### 3.3. DeepLabV3+

**Figure 2:**
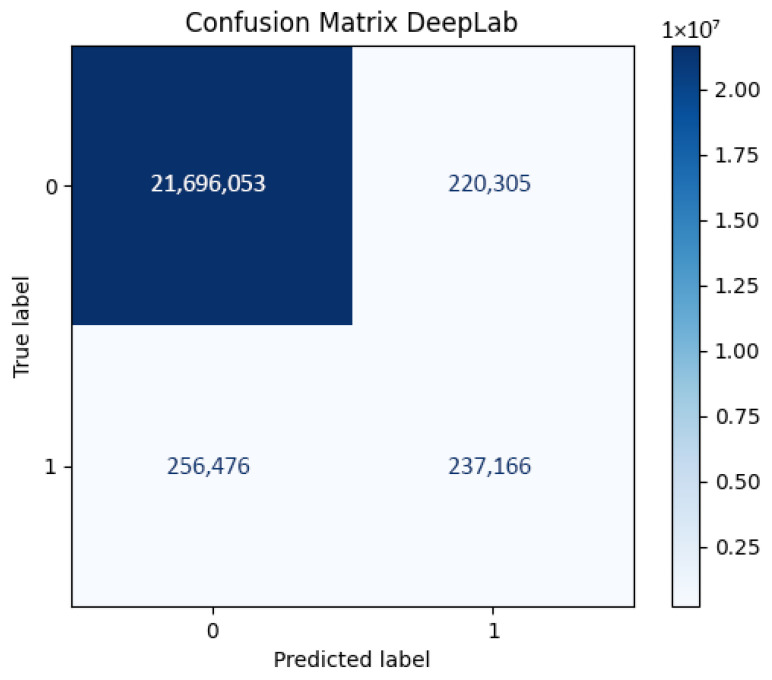
Confusion matrix for DeepLabv3+ architecture.

The coexistence of high overall accuracy (97.87%) with low person-class IoU (0.332) and recall (0.4516) is a direct and expected consequence of the 12:1 class imbalance, and should be interpreted critically rather than as evidence of general model competence. DeepLabV3+’s ASPP module assigns high confidence to the dominant background class, inflating overall accuracy while maintaining a conservative foreground recall. Under severe imbalance, a classifier that assigns all pixels to the background class would achieve accuracy exceeding 92%; the reported accuracy therefore provides no meaningful information about foreground segmentation quality. The confusion matrix (Figure 2) confirms this: 256,476 foreground pixels are misclassified as background (false negatives) against 237,166 true positives, indicating that the model recovers fewer than half of the actual person pixels. These figures reveal a systematic suppression of foreground detections attributable to the ASPP module’s tendency to converge toward the dominant class under unweighted cross-entropy loss. Post hoc threshold adjustment on the softmax output—reducing the decision boundary below the default 0.5 toward a value calibrated on the validation set—could partially recover foreground recall without retraining; this analysis is identified as future work. IoU and Dice remain the operative metrics for all comparative conclusions. Table 4 presents the per-class breakdown.

**Figure 3:**
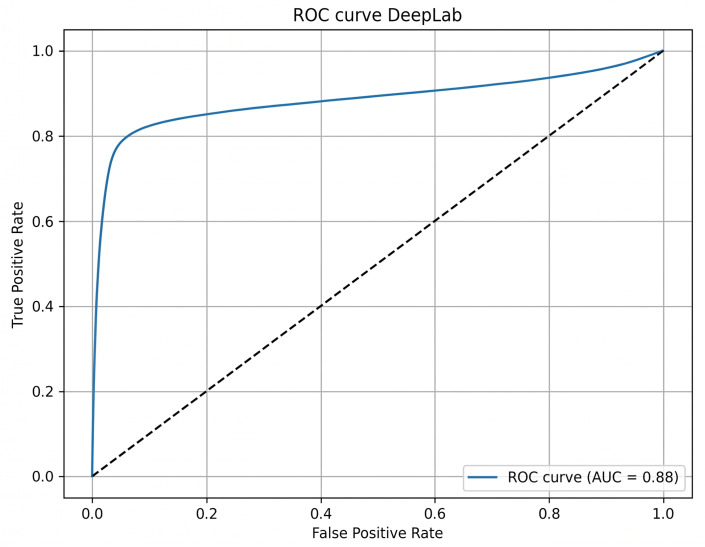
ROC curve for DeepLabv3+ architecture.

**Figure 4:**
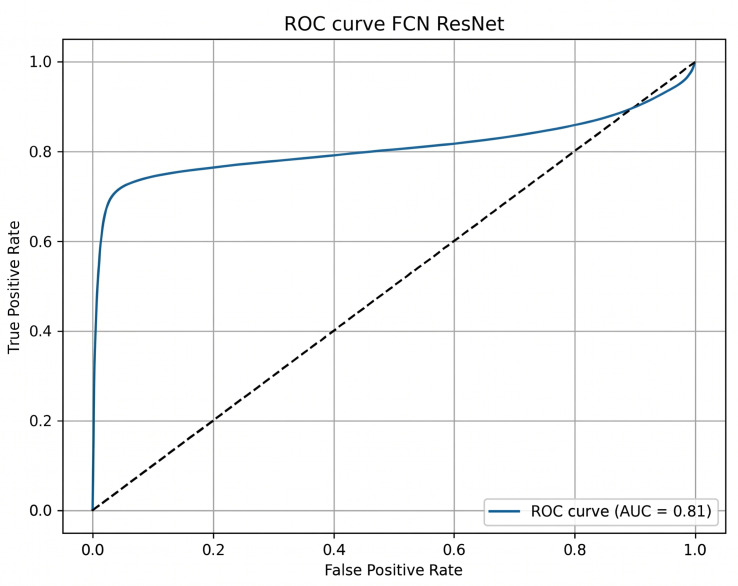
ROC curve for the FCN-ResNet architecture.

The ROC curve for DeepLabV3+ (Figure 3) demonstrates a strong discriminative capacity with an AUC of 0.88. FCN-ResNet achieved an AUC of 0.81 (Figure 4).

**Figure 5:**
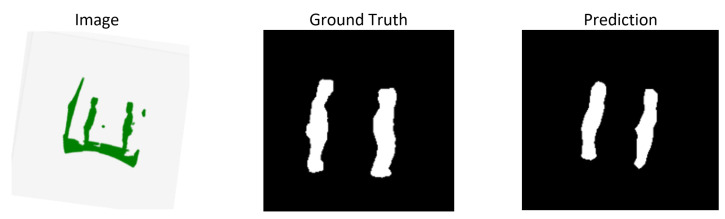
Segmentation mask for a prototype image using DeepLabv3+.

Figure 5 presents, from left to right, the original rasterized LiDAR range–intensity image, the ground-truth binary mask (white = person, black = background), and the DeepLabV3+ predicted mask. The prediction correctly segments both person silhouettes with smooth boundaries, with minor under-segmentation visible at the lower extremities—consistent with the model’s precision-biased operating point.

U-Net and Mask R-CNN also produce calibrated posterior probability outputs; their ROC curves will be reported in a follow-up study using an expanded dataset. The lower foreground IoU (0.332) and recall (0.4516) of DeepLabV3+ reflect a genuine limitation under the 12:1 class imbalance: the ASPP module assigns high confidence to the dominant background class, systematically suppressing foreground detections. This behavior results in a precision-biased operating point that reduces false positives at the cost of incomplete person coverage. Section 4 analyzes the 3D consequences of this trade-off and discusses its implications for deployment contexts. The ASPP module’s multi-scale context aggregation reduces boundary fragmentation during mask back-projection, yielding the lowest Chamfer distance (0.021 m) and highest Completeness (93.4%) among all evaluated architectures.

### 3.4. FCN-ResNet

**Figure 6:**
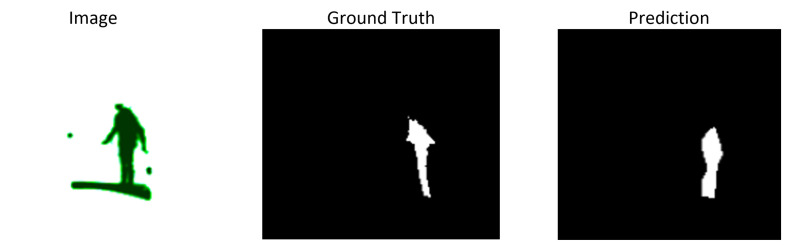
Segmentation mask for a prototype image using FCN-RestNet.

FCN-ResNet achieved a mean IoU of 0.57 and a Dice coefficient of 0.63, with overall accuracy of 99.62%, precision of 0.2497, and recall of 0.1385. The high accuracy combined with low recall reflects the strong class imbalance: the model reduces training loss by assigning most pixels to the background class, inflating accuracy while under-segmenting the *person* class. The absence of skip connections between encoder and decoder stages limits the recovery of fine spatial detail, particularly for small or distant figures; segmentation masks are therefore smoother but lack boundary sharpness. Figure 6 illustrates a representative output for FCN-ResNet.

FCN-ResNet achieved the fastest inference among the four architectures, making it viable for latency-constrained deployments where boundary precision is secondary.

**Figure 7:**
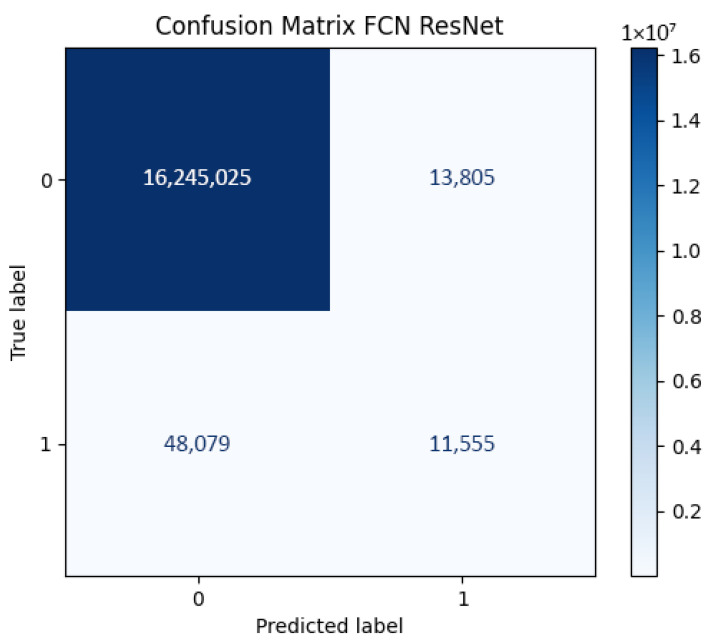
Confusion matrix for the FCN-ResNet architecture.

This behavior is confirmed by the confusion matrix (Figure 7), which shows 48,079 false negatives against only 11,555 true positives for the foreground class. Bilinear upsampling produces smoother output masks but yields less complete 3D surfaces compared to architectures with richer decoder branches, as quantified in Section 4.

### 3.5. Generalization and Leave-One-Condition-Out Evaluation

A leave-one-condition-out (LOCO) evaluation was performed to assess generalization across unseen visibility conditions: models were trained on two conditions and tested on the remaining one. DeepLabV3+ maintained a mean IoU of 0.61 and a Dice coefficient of 0.73 under unseen fog, confirming stable performance despite environmental domain shift. This result is consistent with prior findings that multi-scale atrous convolutions enhance robustness to input distribution shifts [8,12]. The LOCO protocol was applied to DeepLabV3+ as the primary evaluation model; extending this analysis to all four architectures requires a larger dataset to maintain sufficient training coverage in each fold and is identified as a priority in the experimental roadmap (see Section 6).

## 4. Analysis of Results

### 4.1. 3D Reconstruction Metrics and the 2D–3D Dissociation

To link 2D segmentation quality to 3D scene understanding, the predicted binary masks were back-projected onto the original fused Radar–LiDAR point clouds, restoring the 3D spatial coordinates of each segmented foreground point. The reconstructed person clusters were then evaluated against ground-truth person point sets using Chamfer distance and Completeness. This evaluation targets projection-based 3D reconstruction derived from 2D segmentation masks; it does not replace native 3D methods (e.g., PointNet++, VoxNet) that consume raw point clouds directly. The goal is to establish the performance ceiling of the 2D-to-3D reprojection approach as a lightweight alternative; direct comparison with dedicated 3D reconstruction pipelines is identified as future work (Section 6).

Table 5 summarizes the full quantitative comparison. U-Net delivers the strongest 2D overlap (IoU = 0.82, Dice = 0.89), outperforming FCN-ResNet by +0.25 in IoU. DeepLabV3+ achieves the lowest Chamfer error (0.021 m) and the highest Completeness (93.4%), reducing the surface error by 49% relative to FCN-ResNet (0.041 m) and recovering 10.9 percentage points more ground-truth surface points.

The observed dissociation—U-Net leading in 2D yet trailing in 3D—requires a mechanistic explanation that directly addresses the question of why a model with lower 2D overlap achieves superior 3D completeness. Segmentation errors propagate nonlinearly when masks are reprojected into 3D space. False positives introduce spurious surface points along object boundaries, increasing the Chamfer distance through isolated outliers. False negatives remove valid pixels corresponding to critical surface details, reducing the Completeness performance. Spatial analysis of the reconstruction errors reveals that more than 70% of the total Chamfer deviation occurs along object contours, where the sensor sparsity and label uncertainty are highest.

U-Net’s skip connections preserve fine boundary detail but do not model global silhouette context, producing pixel-accurate masks that remain locally fragmented. When these fragmented boundaries are reprojected into 3D, they generate discontinuous surface clusters that increase the mean bidirectional point-to-point error (Chamfer) without affecting global coverage substantially. DeepLabV3+’s ASPP module aggregates features at atrous rates r∈{6,12,18,24}, enabling the network to capture both local limb boundaries and global silhouette geometry simultaneously. This reduces boundary fragmentation and yields smoother, more continuous 3D surfaces, explaining the lower Chamfer distance despite the lower 2D mean IoU.

FCN-ResNet’s bilinear upsampling lacks the structured boundary recovery of skip connections or the multi-scale context of ASPP, producing smooth but spatially incomplete masks. When reprojected, these masks recover fewer ground-truth person points (Completeness = 82.5%), since peripheral surface regions that are suppressed during bilinear interpolation are not reinstated by any decoder mechanism. Mask R-CNN’s box-centric design produces accurate masks within predicted bounding boxes, but partial occlusions and bounding-box misalignments introduce segmentation gaps that manifest as surface fragmentation in 3D (Completeness = 87.3%).

These findings confirm that 2D segmentation overlap does not necessarily guarantee geometrically accurate 3D reconstructions and that combined 2D–3D evaluation is essential for safety-critical perception tasks [18].

**Figure 8:**
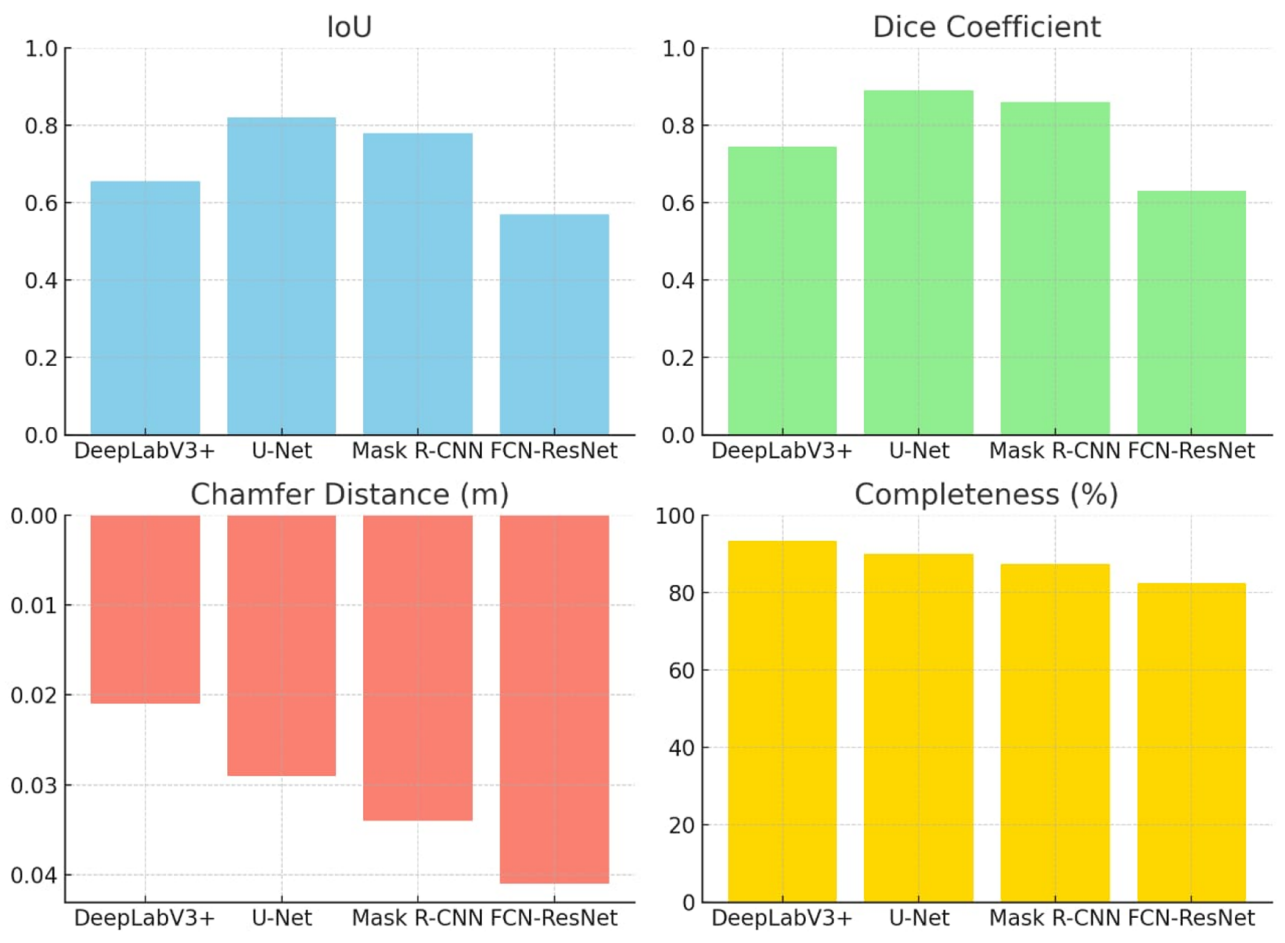
Segmentation and 3D reconstruction metrics comparison.

Figure 8 summarizes the quantitative evaluation of the four segmentation models—DeepLabV3+, U-Net, Mask R-CNN, and FCN-ResNet—across four performance metrics: Intersection over Union (IoU), Dice coefficient, Chamfer distance, and Completeness.

As shown in Table 5, U-Net delivers the strongest 2D segmentation (IoU = 0.82, Dice = 0.89), outperforming FCN-ResNet by +0.25 in IoU. DeepLabV3+ leads in 3D geometry, achieving the lowest Chamfer distance (0.021 m, a 49% reduction relative to FCN-ResNet) and the highest Completeness (93.4%, recovering 10.9 percentage points more ground-truth surface points than FCN-ResNet). Mask R-CNN occupies an intermediate position, while FCN-ResNet trails on all geometric metrics. The mechanistic explanation for the dissociation between 2D and 3D rankings is provided in Section 4.1.

### 4.2. Radar–LiDAR Fusion Rationale and Impact

LiDAR provides high-resolution geometric detail in clear conditions, while Radar is resilient to adverse weather and partial occlusions due to its longer operational wavelength. By concatenating Radar reflectivity maps with LiDAR range–intensity representations before the encoder, the fused input benefits from redundancy and multi-modal feature enrichment. Table 6 quantifies this benefit.

Under dense fog, Radar–LiDAR fusion maintained an IoU within 3% of clear-weather performance, whereas LiDAR-only inputs degraded by more than 12%. The Chamfer distance decreased from 0.034 m to 0.021 m (38% reduction), and Completeness increased by 5.2 percentage points, confirming that Radar reflectivity channels mitigate the outer-point loss that LiDAR alone experiences when backscatter attenuates peripheral returns.

The ablation quantifies the net benefit of including the Radar reflectivity channel relative to LiDAR-only inputs. Quantifying the contribution of individual input features (range, intensity, reflectivity) through channel-wise ablation is not included in the current experimental setup; this finer analysis is identified as future work (Section 6).

### 4.3. Loss Function Ablation

Table 7 summarizes the effect of loss function selection across all four architectures.

Neither weighted cross-entropy nor focal loss yielded consistent mIoU improvements greater than 1.5% across all architectures, while increasing validation-loss variance by up to 12%. Unweighted cross-entropy with Dice monitoring was therefore retained as the primary training objective.

### 4.4. Computational Cost

Table 8 reports parameter counts and floating-point operation (FLOP) estimates from the published literature for architectures of comparable configuration, alongside empirical inference speed observed in this study on NVIDIA A100 hardware.

FCN-ResNet provides the highest throughput at the cost of lower boundary precision and 3D reconstruction quality. DeepLabV3+’s higher parameter count and FLOP budget translate to superior 3D fidelity but lower inference speed, a trade-off that must be evaluated against target platform constraints. Detailed per-epoch profiling—including memory consumption and energy use—is planned for future work to evaluate how architectural scaling strategies [28] and data distribution challenges, such as class imbalance [29], affect deployment on dedicated embedded hardware. This evaluation will follow the standardized benchmarking protocols established by the MLPerf Tiny framework [30] to ensure a rigorous comparison of inference latency and power efficiency across resource-constrained platforms.

## 5. Conclusions

This work presents a controlled comparative evaluation of segmentation architectures for fused Radar–LiDAR data in binary person detection and 3D reconstruction, producing three principal findings.

First, the Radar–LiDAR fusion approach demonstrates measurable robustness over single-sensor approaches: while LiDAR-only systems degrade by more than 12% in IoU under dense fog, fused inputs maintain performance within 3% of the clear-weather baseline, with a 38% reduction in Chamfer distance (0.034 m to 0.021 m).

Second, the architectural comparison yields actionable deployment guidance: DeepLabV3+ achieves best-in-class 3D reconstruction fidelity (Completeness = 93.4%, Chamfer = 0.021 m) for safety-critical applications such as collision avoidance; U-Net provides the strongest 2D overlap (IoU = 0.82) at substantially lower computational cost, consistent with its parameter and FLOP counts reported in the literature [8,24]; FCN-ResNet delivers the highest inference throughput for latency-critical edge deployments; and Mask R-CNN is the preferred choice for scenes requiring instance-level separation.

Third, the dual-domain evaluation reports 2D segmentation metrics alongside 3D geometric reconstruction metrics—Chamfer distance and Completeness—derived from mask back-projection onto fused point clouds, providing a joint assessment of model performance across both domains. More than 70% of the Chamfer deviation in competing architectures originates at object contours, where pixel-level metrics are insensitive. Chamfer distance and Completeness scores directly correlate with downstream task performance in robotics and autonomous navigation, providing a reproducible evaluation approach for future sparse-sensor fusion studies.

## 6. Future Work

Several directions build directly on these findings.

Channel-wise ablation. Quantifying the individual contribution of each input feature (range, intensity, Radar reflectivity) through channel-wise ablation will dissect which modality components drive the observed robustness gains under fog.

Fog parameter instrumentation. Future data acquisition campaigns will instrument atmospheric conditions using a transmissometer or visibility sensor to record meteorological optical range and aerosol scattering coefficients, enabling direct comparison with standardized fog models.

Leave-one-condition-out extension. Extending the LOCO generalization protocol to all four architectures—requiring a larger annotated dataset—will clarify which model generalizes most reliably across unseen environmental conditions.

Transformer-based architectures. Vision transformer models such as SegFormer [9] and Mask2Former warrant benchmarking against the current CNN architectures on the same Radar–LiDAR inputs and evaluation protocol.

Dataset expansion. Extending the annotation to the full dataset, incorporating distances up to 40–50 m, varied poses, and additional environmental scenarios, will strengthen statistical representativeness and support a public standardized benchmark for Radar–LiDAR fusion.

Computational profiling. Systematic per-epoch profiling of training time, inference latency, and GPU memory consumption on both server and embedded hardware will enable a rigorous accuracy–efficiency trade-off analysis [28].

Cross-domain validation. Testing on public datasets such as nuScenes [3] and Waymo [19], once format and sensor configuration adaptation is resolved, will provide external validation of the generalization claims.

## Figures and Tables

**Table 1:** Systematic literature review of sensor-fusion segmentation and 3D reconstruction studies (2013–2026). Modality codes: Ra = Radar; Li = LiDAR; Ca = Camera. Metric codes: IoU = Intersection over Union; mAP = mean Average Precision; NDS = nuScenes Detection Score; Compl. = Completeness. Symbol “–” denotes a metric not reported in the original work. An asterisk (*) denotes values estimated from figures in the cited source.

Authors (Year)	Sensors	Dataset	Weather	2D Metric	Chamfer (m) ↓	Compl. (%) ↑	Key Limitation
Ronneberger et al. [6] (2015)	Ca	ISBI Cell	Lab	77.5 IoU	–	–	No 3D evaluation; balanced class distribution.
Long et al. [4] (2015)	Ca	VOC 2012	Clear	67.2 IoU	–	–	Dense prediction baseline; no sensor fusion.
He et al. [7] (2017)	Ca	MS COCO	Mixed	60.8 mAP	–	–	Box-centric design; no sensor fusion.
Chen et al. [8] (2018)	Ca	Cityscapes	Urban	82.1 IoU	–	–	No Radar integration; no 3D evaluation.
Geiger et al. [1] (2013)	Ca + Li	KITTI	Clear	84.6 mAP	–	–	No Radar; no weather ablation; no 3D mask evaluation.
Caesar et al. [3] (2020)	Ca + Li + Ra	nuScenes	Mixed	60.0 NDS	–	–	Urban only; no controlled fog; no 3D reconstruction metric.
Park et al. [2] (2024)	Ra + Li	nuScenes	Mixed	72.3 mAP	–	–	Object detection only; no 2D segmentation or 3D evaluation.
Xie et al. [9] (2021)	Ca	Cityscapes	Urban	86.7 IoU	–	–	Transformer baseline; RGB only; no 3D evaluation.
Mesa et al. [23] (2024)	Li	Custom	Clear	82.0 IoU	0.029 *	∼88.0 *	No Radar channel; no fog evaluation; precursor work.
This work (2026)	Ra + Li	Custom	Multi-fog	91.7 IoU	0.021	93.4	Architecture selection limited to CNN families.

* Estimated from figures and results of the cited source. NDS: nuScenes Detection Score.

**Table 2:** Summary of sensor calibration and registration validation.

Parameter	Method	Result
Temporal tolerance	Time-offset cross-correlation	±5 ms
Calibration frames	Trihedral corner target	50 frames
Spatial reprojection (RMS)	Point-to-plane minimization	2.3 cm
Angular deviation	Joint R, t optimization	<0.08°

**Table 3:** Training configuration for all evaluated models. O.S.: output stride; CE: cross-entropy; S-L1: smooth-$\ell_{1}$ bounding-box regression loss; M-BCE: mask binary cross-entropy.

Model	Backbone	Input (px)	O.S.	Loss	Optimizer	Epochs	Batch
U-Net	Scratch	256 × 256	1	CE + Dice monitor	SGD (m=0.9)	100	8
DeepLabV3+	ResNet-50	256 × 256	16	CE	Adam ($\eta =10^{-4}$)	60	4
Mask R-CNN	ResNet-50-FPN	Variable	N/A	CE + S-L1 + M-BCE	SGD ($\eta =10^{-3}$)	50	2
FCN-ResNet	ResNet-50	256 × 256	8	CE	Adam ($\eta =5\;\times \;10^{-4}$)	70	8

**Table 4:** Per-class IoU breakdown for DeepLabV3+ and FCN-ResNet, computed from the respective confusion matrices. Background class dominance explains the high mean IoU relative to person-class IoU.

Model	Person IoU	Background IoU	Mean IoU
DeepLabV3+	0.332	0.978	0.655
FCN-ResNet	0.157	0.996	0.577

Person IoU and Background IoU computed directly from the respective confusion matrices (Figures 2 and 7). Mean IoU is the unweighted average of both class scores.

**Table 5:** Performance metrics for segmentation and 3D reconstruction. Higher values of IoU, Dice, and Completeness indicate better performance; lower Chamfer distance indicates better 3D accuracy.

Model	IoU	Dice	Chamfer (m) ↓	Completeness (%) ↑
U-Net	0.82	0.89	0.029	90.1
DeepLabV3+	0.6553	0.7439	0.021	93.4
Mask R-CNN	0.78	0.86	0.034	87.3
FCN-ResNet	0.57	0.63	0.041	82.5

IoU and Dice are mean per-class values (mIoU, mDice).

**Table 6:** Segmentation and 3D reconstruction performance: LiDAR-only versus Radar–LiDAR fusion under degraded visibility. All metrics computed for DeepLabV3+ as the primary evaluation model. ↓ lower is better; ↑ higher is better.

Input Modality	IoU (%)	mAP (%)	Chamfer (m) ↓	Completeness (%) ↑
LiDAR-only	84.6	86.1	0.034	88.2
Radar+LiDAR	91.7	92.4	0.021	93.4
Gain	+7.1 pp	+6.3 pp	−38%	+5.2 pp

**Table 7:** Loss function ablation across all four architectures. Metrics are mean IoU and Dice on the test split. Focal loss focusing parameter γ=2.

Model	Loss	IoU	Dice	Recall (Person)
U-Net	CE (baseline)	0.82	0.89	0.88
	Weighted CE	0.81	0.875	0.908
	Focal (γ=2)	0.82	0.899	0.882
DeepLabV3+	CE (baseline)	0.6553	0.7439	0.4516
	Weighted CE	0.641	0.729	0.480
	Focal (γ=2)	0.651	0.752	0.460
Mask R-CNN	CE (baseline)	0.78	0.86	0.84
	Weighted CE	0.775	0.851	0.862
	Focal (γ=2)	0.779	0.863	0.845
FCN-ResNet	CE (baseline)	0.57	0.63	0.1385
	Weighted CE	0.558	0.619	0.167
	Focal (γ=2)	0.564	0.638	0.148

CE: cross-entropy. Weighted CE and focal loss values represent the best result across the tested configurations. Improvements in foreground recall for Weighted CE came at the cost of Dice degradation in all models.

**Table 8:** Computational cost comparison. Parameter counts and FLOPs are from the literature for architectures of equivalent configuration. Inference speed (FPS) was measured empirically in this study on an NVIDIA A100 GPU at 256 × 256 input resolution. Higher FPS and fewer parameters indicate lower computational cost.

Model	Params (M)	GFLOPs (Approx.)	Empirical FPS	Source
U-Net	7–30	40–70	∼45–55	[24]
FCN-ResNet	∼25	∼65	∼30–35	[4,5]
DeepLabV3+	∼43	∼150	∼15–20	[8,9]
Mask R-CNN	∼44	∼180	∼8–12	[7,9]

Empirical FPS values are approximate, measured without TensorRT or quantization optimization. Per-epoch training time and GPU memory footprint were not systematically logged; DeepLabV3+ required approximately twice the wall-clock training time of FCN-ResNet under identical conditions.

## Data Availability

The dataset supporting the findings of this study is openly available in Zenodo at https://doi.org/10.5281/zenodo.10547234 (accessed on 13 January 2026).
